# Surgical Treatment of Trochlear and Abducens Nerve Palsies

**Published:** 2010-01

**Authors:** David Coats

**Affiliations:** Texas Children’s Hospital, Baylor College of Medicine, Houston, Texas, USA

This issue of the *Journal of Ophthalmic and Vision Research* contains two reports summarizing the 10 year surgical treatment experience of trochlear and abducens nerve palsies at Labbafinejad Medical Center, a large tertiary referral practice in Tehran, Iran.^1^,^2^ Generally, the authors achieved an excellent overall success rate in terms of alignment in primary position, with the majority of patients requiring only one operation. Based on personal experience and the reported literature,^3^,^4^ I was surprised that diplopia and anomalous head position were infrequent chief complaints. In my practice, anomalous head position is the most common complaint in children while diplopia is the most common complaint in adults. In these reports however, diplopia was the chief complaint in only 26% and 21.2% of patients with trochlear and abducens nerve palsy respectively; corresponding figures for abnormal head posture were only 9.6% and 3%. Even with a large number of very young patients in the abducens palsy group, this seems unusual and perhaps can be explained by regional differences in the timing of presentation, patient understanding, expectations, needs, and/or other factors.

Both reports present a heterogeneous group of patients on which a variety of operative approaches were utilized. It would have been interesting to see more information on why the authors preferred one surgical approach over another and additional information on outcomes other than alignment, though I understand this can be difficult to provide in a retrospective study. Without these additional data, it would be difficult to judge the merits of one surgical procedure over another.

In the report on trochlear nerve palsy, the authors stress the importance of surgically treating larger than concurrent horizontal deviations, verifying prior literature. I tend to identify inferior oblique over-action more frequently and superior oblique under-action less commonly in my trochlear nerve palsy patients than these authors report. Also, patients with unilateral and bilateral trochlear nerve palsies typically have very different prognosis with surgery, therefore reporting bilateral cases separately, rather than grouping all patients together would have been of value.

The rate of congenital abducens palsy requiring surgical correction was remarkably high at almost 20% as compared both to personal experience and published literature; perhaps this may be the result of the tertiary nature of the authors’ practice or regional factors. I cannot explain why the abducens nerve palsy patients had such small anomalous head positions (mean 15 degrees or less for all treatment groups) in the face of very large preoperative deviations (mean 50.3 PD). I question the authors’ statement that “… strabismus surgery has a self-adjustable nature … (with) greater response with more severe strabismus.” All patients in the transposition group had complete absence of lateral rectus function and thus were a homogeneous group in this regard and those in the non-transposition group presumably underwent horizontal strabismus surgery titrated to the size of their deviation.

In summary, these authors have reported on the treatment of large groups of patients with trochlear and abducens palsies with good to excellent success in terms of final deviation in primary position after completion of treatment. More details about the surgical paradigm and other outcome parameters would have been useful to help put these reports in perspective relative to published literature on these topics.
